# 

**Published:** 1860-04

**Authors:** 


No. XI. APRIL—1860. Vol. XV.

### NEW YORK MONTHLY REVIEW

OF

### Medical & Surgical Science,

AND

### BUFFALO MEDICAL JOURNAL.

EDITED BY AUSTIN FLINT, Jr., M.D.,

Prof. of Physiology and Microscopic Anatomy in the New York Medical College.

And J. H. DOUGLAS, M.D.

NEW YORK:

CHARLES B. NORTON, APPLETON'S BUILDING,

346 AND 348 BROADWAY.

*LONDON:* H. BAILLIERE, 219 REGENT STREET. *PARIS:* J. B. BAILLIERE ET FILS, RUE HAUTEFEUILLE. *MADRID:* C. BAILLY-BAILLIERE, CALLE DEL PRINCIPE. *LEIPZIG:* BERNARD HERMANN, AGENT OF B. WESTERMANN & CO.

Hall, Clayton & Co., Printers, 46 Pine St., N. Y.

Price $3.00 a Year, payable in Advance.

